# Specific EtMIC3-binding peptides inhibit *Eimeria tenella* sporozoites entry into host cells

**DOI:** 10.1186/s13567-020-00873-y

**Published:** 2021-02-17

**Authors:** Wenjing Chen, Chunli Ma, Guanghao Li, Zhipeng Jia, Xuelian Yang, Xinghui Pan, Dexing Ma

**Affiliations:** 1 College of Veterinary Medicine, Northeast Agricultural University, NO. 600 Changjiang Road, Xiangfang District, Harbin, Heilongjiang 150030 China; 2 Heilongjiang Key Laboratory for Experimental Animals and Comparative Medicine, Harbin, Heilongjiang 150030 China; 3 College of Food Science, Northeast Agricultural University, Harbin, Heilongjiang 150030 China

**Keywords:** EtMIC3, sporozoites, invasion, protective effects, molecular docking

## Abstract

Avian coccidiosis caused by *Eimeria* leads to huge economic losses on the global poultry industry. In this study, microneme adhesive repeat regions (MARR) bc1 of *E. tenella* microneme protein 3 (EtMIC3-bc1) was used as ligand, and peptides binding to EtMIC3 were screened from a phage display peptide library. The positive phage clones were checked by enzyme-linked immunosorbent assay (ELISA). Competitive ELISA was applied to further verify the binding capability between the positive phages and recombinant EtMIC3-bc1 protein or sporozoites protein. The inhibitory effects of target peptides on sporozoites invasion of MDBK cells were measured in vitro. Chickens were orally administrated with target positive phages and the protective effects against homologous challenge were evaluated. The model of three-dimensional (3D) structure for EtMIC3-bc1 was conducted, and molecular docking between target peptides and EtMIC3-bc1 model was analyzed. The results demonstrated that three selected positive phages specifically bind to EtMIC3-bc1 protein. The three peptides A, D and W effectively inhibited invasion of MDBK cells by sporozoites, showing inhibited ratio of 71.8%, 54.6% and 20.8%, respectively. Chickens in the group orally inoculated with phages A displayed more protective efficacies against homologous challenge than other groups. Molecular docking showed that amino acids in three peptides, especially in peptide A, insert into the hydrophobic groove of EtMIC3-bc1 protein, and bind to EtMIC3-bc1 through intermolecular hydrogen bonds. Taken together, the results suggest EtMIC3-binding peptides inhibit sporozoites entry into host cells. This study provides new idea for exploring novel strategies against coccidiosis.

## Introduction

The phylum apicomplexa includes several well-known unicellular protozoan parasites such as *Plasmodium*, *Toxoplasma*, *Neospora* and *Eimeria*, all of which infect human or animals. *Eimeria tenella* is one of the seven *Eimeria* species, colonizes caecal epithelium cells and causes avian coccidiosis that leads to enormous economic losses to the global poultry industry [1]. Considering the emergence of drug-resistant parasites [2, 3], alternative measures against coccidiosis are urgently required. With the aim of developing novel anti-coccidial strategies, researchers gradually focus on the mechanism for *Eimeria* invasion of host cells. *E. tenella* microneme proteins are secreted on the surface of parasites by microneme organelles, and could be recognized by receptors located on the surface of the host cells. Microneme proteins play a crucial role in invasion of host cells by *Eimeria* parasites. Several microneme proteins have already been reported, including EtMIC1 [4], EtMIC2 [5, 6], EtMIC3 [7, 8], EtMIC4 [9, 10], and EtMIC5 [11]. EtMIC3 contains seven tandem microneme adhesive repeat regions (MARR), named EtMIC3-MARa, EtMIC3-MARb, EtMIC3-MARc and EtMIC3-MARd, among which MARc consists of four repeated domains (MARc1, MARc2, MARc3 and MARc4). MARb, MARc and MARd were reported to actively bind with host cells [7]. Previous studies demonstrated that EtMIC3 effectively facilitates the invasion of sporozoites into host cells by specifically recognizing sialylated glycans, and is responsible for guiding *E. tenella* sporozoites to the invasion sites in chicken gut [12, 13]. Taking into account that EtMIC3 protein plays key roles in the process of sporozoites invasion of host cells, we postulated that peptides specifically binding to EtMIC3 protein could effectively inhibit invasion of cells by *E. tenella* sporozoites. With the aim to verify the above hypothesis and explore novel strategies against coccidiosis, in the present study, the recombinant MARb and one of the four repeated MARc domains of EtMIC3 protein (EtMIC3-bc1) were selected as ligand, and peptides binding to EtMIC3 were screened from phage display peptide library. Then the effects of target phages or corresponding peptides on inhibiting sporozoites invasion of cells were measured in vivo and in vitro. Furthermore, the model of three-dimensional (3D) structure for EtMIC3-bc1 protein was conducted, and molecular docking between target peptides and EtMIC3-bc1 model was analyzed.

## Materials and methods

### Expression and purification of EtMIC3-bc1 protein

**Figure 1:**
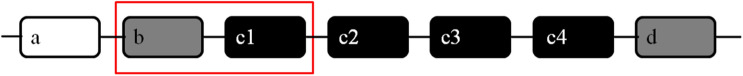
**Schematic illustration of EtMIC3 protein.** *E. tenella* microneme proteins 3 (EtMIC3) contains seven tandem microneme adhesive repeat regions (MARR), and were named EtMIC3-MARa, EtMIC3-MARb, EtMIC3-MARc, and EtMIC3-MARd, respectively. MARc consists of four repeated domains (MARc1, MARc2, MARc3, MARc4).

The objective fragment consists of MARb and MARc1 of EtMIC3 (EtMIC3-bc1) (Figure 1) was cut off from pUC-EtMIC3-bc1 plasmid (stored in our lab) by restriction enzyme *Bam*H I and *Xho* I. The 864 bp fragment of EtMIC3-bc1 was subcloned into pET30a vector (Novagen, Madison, WI) to generate positive plasmid pET30a-EtMIC3-bc1. The above plasmid was transformed into *E. coli* BL21 bacteria to produce recombinant positive bacteria. The bacteria culture was induced by isopropyl-b-D-thiogalactopyranoside (IPTG), and then sonicated (300 W for 3 s, with 3 s interval). The purification of EtMIC3-bc1 protein was performed by affinity chromatography with Ni-conjugated sepharose as previously described [14]. The concentration of purified EtMIC3-bc1 protein was determined, and then separated by 12% SDS-PAGE. The expected protein bands were visualized using Coomassie brilliant blue stain R-250 (Beyotime, China).

### Preparation of antisera against EtMIC3-bc1 protein

The polyclonal antisera against EtMIC3-bc1 protein was prepared according to previous report with some modifications [15]. Briefly, 7-week-old specific pathogen-free (SPF) New Zealand White rabbit was subcutaneously injected with 2.0 mg of purified EtMIC3-bc1 protein (1 mg/mL) emulsified with the same volume of Freund’s complete adjuvant (Sigma, USA) around neck, inguinal and axillary lymph nodes. After two weeks, the immunization was boosted with 1.0 mg of purified EtMIC3-bc1 protein emulsified with the same volume of Freund’s incomplete adjuvant (Sigma, USA). Another two booster immunizations were performed at one week interval. On day 7 post the last immunization, blood was sampled via the marginal ear vein, and sera were collected and stored at − 20 °C until use. The titers of the prepared polyclonal antisera against EtMIC3-bc1 protein were tested by indirect enzyme-linked immunosorbent assay (ELISA) as previously described [16]. The specificity of the prepared polyclonal antisera against EtMIC3-bc1 protein was detected by western blot. Briefly, *E. tenella* sporozoites were purified and sonicated as previously described [14], and protein samples were separated by SDS-PAGE, then transferred to nitrocellulose membranes. The membranes were incubated with rabbit anti-EtMIC3-bc1 polyclonal antisera for 2 h. After washing with TTBS (0.1% Tween 20, 50 mmol/L Tris–HCl, 150 mmol/L NaCl, pH 7.5), the membranes were incubated with horseradish peroxidase (HRP)-conjugated goat anti-rabbit IgG antibody (Sigma, USA). The immune complexes were visualized using ECL chemiluminiscence detection kit (Sangon Biotech Co., Ltd, Shanghai,China) according to the provided instructions.

### Screening of phages bind to EtMIC3-bc1

The screening of peptides binding to EtMIC3-bc1 protein was carried out as described in previous reports [14, 17] with some modifications based on phage display peptide library kit (New England Biolabs, USA). Briefly, 96-well plates were coated with 100 μL per well of purified EtMIC3-bc1 protein (100 μg/mL), and incubated overnight at 4 ℃. After blocking with 2% BSA for 2 h, the plates were washed with TBS-T (Tris buffered saline with 0.1% Tween-20, pH 7.4) 6 times. The coated protein in each well was reacted with 100 μL of diluted (1:100) phage display peptide library (1.5 × 10^14^ pfu) (New England Biolabs, USA) for 30 min at room temperature. The plates were firstly washed with 0.1% TBS-T for six times, then washed with 0.2 mol/L glycine–HCL (pH 2.2), and ultimately neutralized with 1.0 mol/L Tris–HCL (pH 9.1). The eluent from plates, called the first round of biopanning phage, was titrated and amplified in *E. coli* strain ER2738 according to the protocol. The amplified target phage was precipitated by 20% PEG8000/NaCl (w/v) (2.5 M NaCl), and titrated again according to the protocol. The next three rounds of biopanning were carried out as described above except that TBS-T (TBS with 0.5% Tween-20, pH 7.4) was applied to wash off the unbound phages. The fourth round of biopanning phages were amplified in *E. coli* strain ER2738 based on the recommended protocol in phage display peptide library Kit (New England Biolabs, USA). The amplified phages were stored at 4 ℃ until use.

### Enzyme-linked immunosorbent assay (ELISA)

To further detect the binding capability between the screened phages and EtMIC3-bc1 protein, enzyme-linked immunosorbent assay (ELISA) was performed as previously described [14, 18]. Briefly, the plates coated with 100 μL per well of EtMIC3-b1c protein (100 μg/mL) were incubated overnight, then washed with PBS-T (0.5% Tween-20) and blocked with 5% skim milk. After washing, each well was incubated with 100 μL of target phages (1.0 × 10^12^ pfu). The washed plate was incubated with 100 μL per well of diluted anti-M13 rabbit polyclonal antibody (1:1000). After washing, the plate was incubated with 100 μL per well of diluted horseradish peroxidase (HRP)-conjugated goat anti-rabbit IgG (Sigma, USA) (1:5000), reacted with substrate solution (0.01% H_2_O_2_, 1 mg/mL o-phenylenediamine), and stoped with 2 M sulfuric acid. The optical density at 490 nm (OD490) was recorded by microplate reader (Bio-Rad, USA). Unrelated phages bound to G protein of porcine Vesicular Stomatitis Virus (VSV) (stored in our lab) were used as negative controls. The positive phages that specifically binding to EtAMA1 protein were used as positive control [14]. Each sample was tested in triplicate.

### Sequence analysis

Phage DNA was extracted according to the provided method in M13 isolation kit (BioTeke Corporation Co., Ltd, China), and was used as template to amplify the fragment that contained target peptide using specific primers pair (New England Biolabs). The above DNA fragment was sequenced, and amino acid sequences were deduced from the sequences of nucleotides. The target peptides were synthesized by GenScript Co., Ltd (Nanjing, China).

### Binding capability between EtMIC3-bc1 protein and target phages

To verify the binding capability between the selected phages and EtMIC3-bc1 protein, ELISA and competitive ELISA was performed as previously described [14] with some modifications. Briefly, 96-well plates were coated with three selected pahges (called A, D and W) that serially diluted from 1 × 10^6^ to 1 × 10^12^ pfu, incubated overnight, blocked with 5% skim milk, washed by 0.5% PBS-T, and then incubated with 100 μL per well of purified EtMIC3-bc1 protein (100 μg/mL). After washing, the plate was incubated with rabbit anti-EtMIC3 polyclonal antisera (60 μg/mL) diluted at 1:1000, reacted with 100 μL of HRP-labeled goat anti-rabbit IgG (1:5000) (Boster, Wuhan, China). After incubating for 1 h at 37 ℃, the OD490 values were measured. Each sample was tested in triplicate. The unrelated phages from G protein of VSV (stored in our lab) and the positive phages binding to EtAMA1 protein was used as negative and positive control, respectively.

For competitive ELISA, 50 μL of rabbit anti-EtMIC3 polyclonal antisera diluted at 1:1000, 1:1500, 1:2000 and 1:2500 (with concentrations of 60, 40, 30, 24 and 20 μg/mL, respectively) and 50 μL of EtMIC3-bc1 protein (100 μg/mL) were designed as binding competitors to 100 μL of coated positive phages (1.0 × 10^12^ pfu) in each well of the plate. After incubation, the OD490 values were measured, and each sample was tested in triplicate. The negative and positive phages mentioned above were used as controls.

### Binding capability between sporozoites protein and target phages

*E. tenella* sporozoites were purified by percoll density gradient as described in previous report [14]. Briefly, sporulated *E. tenella* oocysts were broken by shaking with 3 mm diameter glass beads, and the released sporocysts were pooled by centrifugation in 50% Percoll (Sigma-Aldrich). The collected sporocysts were incubated with PBS solution containing 0.25% (w/v) Trypsin, 4% (w/v) taurodeoxycholic acid and 10 mM MgCl_2_. The excysted sporozoites were suspended in 55% Percoll (Sigma-Aldrich), and the sporozoites were harvested after centrifugation. The binding capability between sporozoites protein and target phages were detected by ELISA as described above except that 100 μL sonicated (300 W for 5 s, with 5 s interval) sporozoites (1.0 × 10^6^/mL) protein (1.2 mg/mL) was added to react with coated target phages. The OD490 values were measured. Each sample was tested in triplicate. The negative and positive phages mentioned above were used as controls.

### In vitro effects of synthesized peptides in inhibiting sporozoites invasion into cells

The maximum non-cytotoxic concentration of synthesized peptides A, D and W to MDBK cells was determined using MTT dye 3-(4, 5-dimethylthiazol-2-yl)-2, 5-diphenyltetrazoliumbromid as previously described [14]. The effects of synthesized peptides in inhibiting sporozoites invasion into MDBK cells were carried out according to the reported methods [14, 19]. Briefly, 3 × 10^5^ sporozoites were labelled with carboxyfluorescein diacetate succinimidyl ester (CFDA-SE) (Beyotime, China). The labelled sporozoites were pre-incubated with 100 μL target peptides with final concentrations of 25, 50, 75, 100 and 125 μg/mL at 37 ℃ for 2 h. The incubated solutions were centrifuged to collect sporozoites which were then added to monolayer MDBK cells (1 × 10^5^ per well in 24-well plate) for 10 h at 37 ℃. After 3 washes, cells were digested with 0.25% (w/v) trypsin, and centrifuged to collect pellets which were resuspended in 500 μL PBS (pH 7.2). The MDBK cells invaded by CFDA-SE-labelled sporozoites showing green fluorescent were counted by flow cytometry (Beckman Coulter), and the invasion ratio was calculated. The fluorescently-labelled sporozoites incubating with 100 μL of polyclonal antisera against EtMIC3-bc1 protein (300 μg/mL) (determined in preliminary test) were designed as positive controls. The fluorescently-labeled sporozoites incubating with MDBK cells were used as negative controls.

### In vivo anticoccidial effects provided by target phages

10-day-old specific pathogen-free (SPF) White Leghorn chickens were randomly divided into 5 groups of 15 chicks each. All chickens were individually housed in hanging cages. At 21 days of age, all chickens except in group 1 (non-challenged control group) were orally inoculated with 1 × 10^4^ *E. tenella* sporulated oocysts. At days 0, 1 and 2 after challenging, chickens in group 2 were orally fed 1 × 10^12^ pfu phages A, group 3 with phages D, group 4 with phages W, and group 5 (challenged control group) with PBS (pH 7.2). All animal experiments were complied with the rules of Animal Experiment Ethic Committee of Northeast Agricultural University, China. Chickens in each group were weighed before challenging and at days 7 after challenging to calculate the body weight gain (BWG) [18]. Lesion scores in ceca of chickens (n = 5) from all the groups were determined on day 7 post challenging as previous described [20]. Fecal samples from each chicken within each group between days 7 and 11 post challenging were gathered to count the oocyst output per gram (OPG), and oocyst reduction ratio was calculated as decribed by Lillehoj et al. [21]. Briefly, oocyst reduction ratio = (number of oocysts from challenged control chickens—orally administrated chickens)/ challenged control chickens × 100%.

### Homology modeling and molecular docking

The three-dimensional (3D) structure of EtMIC3-bc1 protein was generated according to the published crystal structures of EtMIC3-MAR1b (PDB code 2LBO) using Swiss-Model (http://swissmodel.expasy.org). The software Discovery Studio 2.5 was used to optimize the 3D structure of EtMIC3-bc1, and the model quality was evaluated as previously described [22]. The tertiary structures of target peptides A, D and W were produced by Swiss-Model. The software Autodock 4.2 and Discovery Studio 2.5 were applied to explore the molecular docking between target peptide and constructed 3D structure of EtMIC3-bc1 protein.

### Statistical analysis

Data was subjected to one-way analysis of variance (ANOVA), and was expressed as means ± SD. ANOVA Duncan’s multiple-comparison procedure was used to compare differences between mean values. Results were considered as significant difference at *P* value less than 0.05.

## Results

### Purification of EtMIC3-bc1 protein

The expression and purification of EtMIC3-bc1 protein was displayed in Additional file 1, showing an expected protein band of 41 kDa.

### Characterization of polyclonal antisera against EtMIC3-bc1 protein

Western blot detection showed that the prepared antisera against EtMIC3-bc1 protein reacted with sporozoites protein and recombinant EtMIC3-bc1 protein, showing protein bands of 41 KDa (Additional file 2). The titer of prepared antisera was 1:2^16^.

### Screening of phages binding to EtMIC3-bc1

The plaque forming unit per milliliter (pfu/mL) of the positive phages binding to EtMIC3-bc1 protein were identified after four rounds of affinity screening (Table 1), demonstrating that the enriched phages bind to EtMIC3-bc1 protein.

**Table 1:** **Titer of phages after four rounds of biopanning and amplification.**

	Rounds of biopanning			
	First	Second	Third	Fourth
Input phage (pfu/mL)	1.5 × 10^11^	1.5 × 10^11^	1.5 × 10^11^	1.5 × 10^11^
Output phage (pfu/mL	6.4 × 10^4^	6.3 × 10^4^	2.3 × 10^5^	8.6 × 10^5^
Amplified phage (pfu/mL)	3.0 × 10^11^	1.3 × 10^11^	4.5 × 10^12^	5.7 × 10^12^

### Characterization of screened phages binding to EtMIC3-bc1

**Figure 2:**
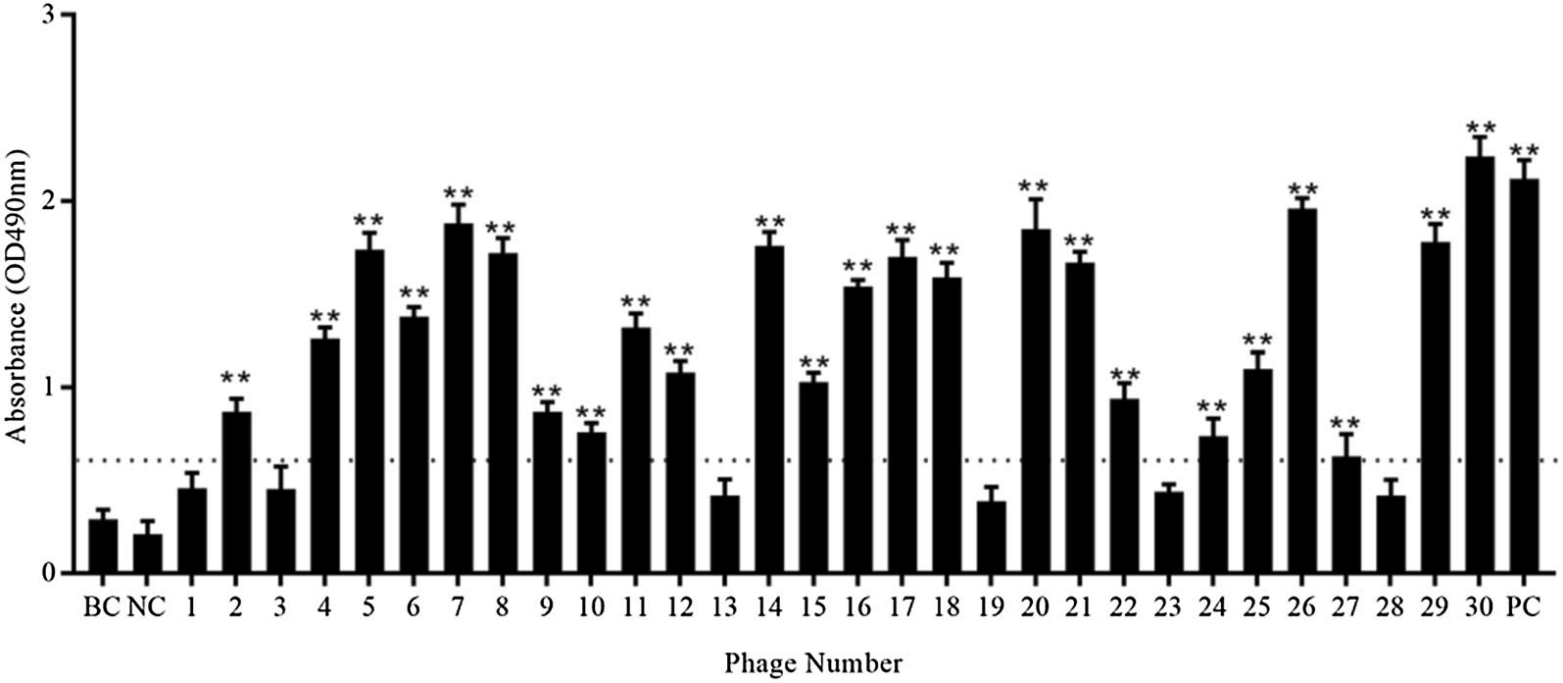
**Detection of binding capability between selected phages amd EtMIC3-bc1 protein.** EtMIC3-bc1-coated plate was incubated with thirty selected phages. The washed plate was then successively incubated with rabbit anti-M13 polyclonal antibody and horseradish peroxidase (HRP)-conjugated goat anti-rabbit IgG. Then substrate solution (0.01% H_2_O_2_ and 1 mg/mL o-phenylenediamine) was added, and the reaction was stopped with 2 M sulfuric acid. The values of optical density (OD) at 490 nm was recorded. The blank control (BC), the negative control (NC) phages from G protein of porcine Vesicular Stomatitis Virus (VSV) (stored in our lab), and the positive control (PC) phages binding to EtAMA1 protein (stored in our lab) were used. Each sample was tested in triplicate. Values represent mean ± SD. Twenty-four selected phages clones showed higher binding capability than other clones (with the number of 1, 3, 13, 19, 23, 28), blank control and netative control phages (*P* < 0.01).  ∗  ∗ *P* < 0.01.

As displayed in Figure 2, among the selected thirty clones, twenty-four phages (except for NO.1, 3, 13, 19, 23 and 28) showed significantly higher binding capability to EtMIC3-bc1 protein than other and control phages (*P* < 0.01).

### Sequencing of positive clones

After sequencing of 24 positive phages clones, 8 peptides with different amino acid composition were observed. The sequences of AGRLLTPTMSLV (peptide A), DYHDPSLPTLGK (peptide D), and WKDVHKAWLLEP (peptide W) appeared 8, 6 and 3 times, respectively.

### Binding assays

**Figure 3:**
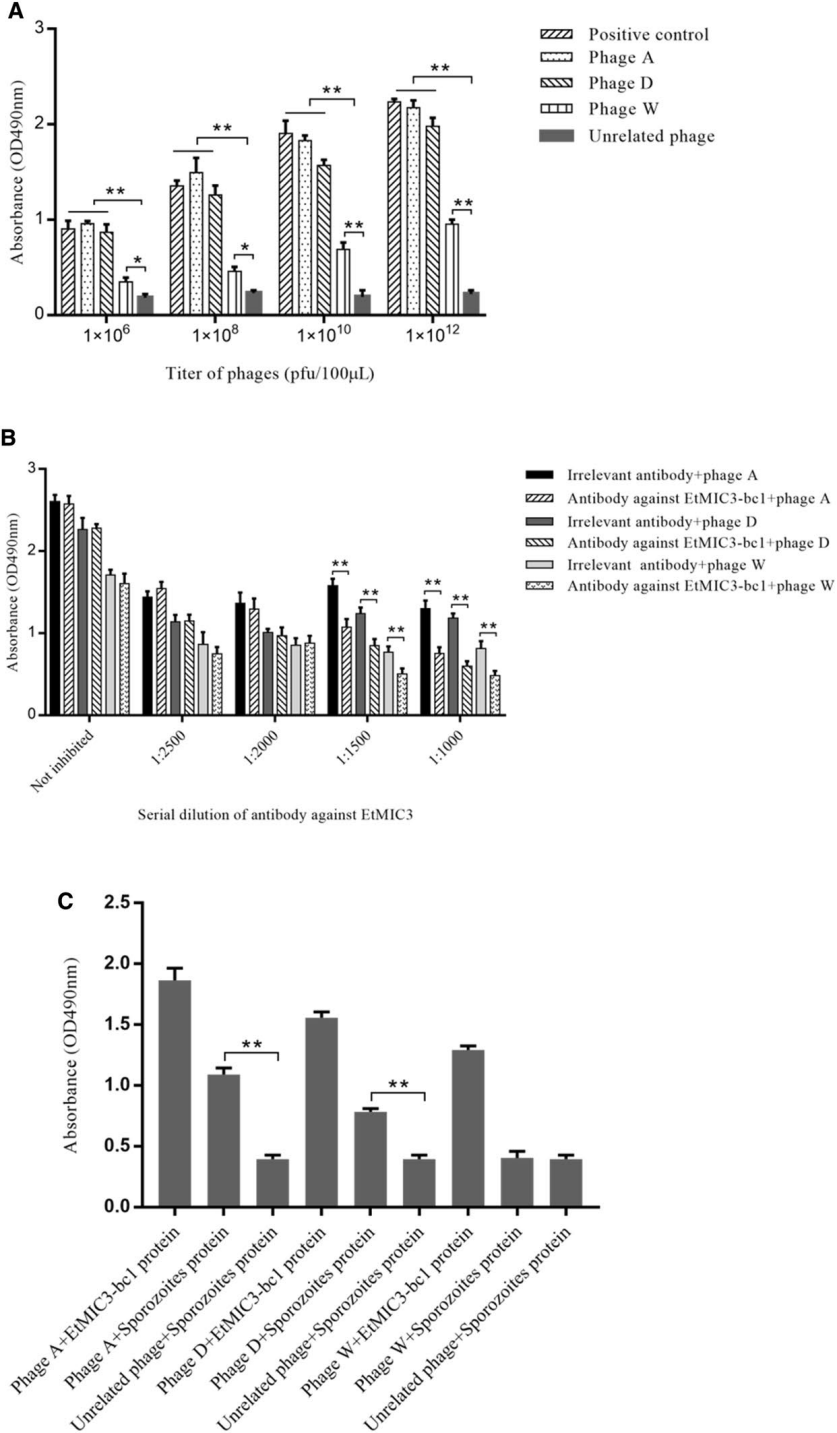
**Binding detection of three target phages with recombinant EtMIC3-bc1 protein and sporozoites protein.** ELISA (**A**,** C**) and competitive ELISA (**B**) were applied to assay the binding capability of three phages A, D and W with recombinant EtMIC3-bc1 protein and sporozoites protein. The phages A, D and W diluted from 1.0 × 10^6^ to 1.0 × 10^12^ pfu were coated in 96-well plate, respectively, and the three phages showed higher binding capability to EtMIC3-bc1 protein than the unrelated phages from G protein of Vesicular stomatitis virus (VSV) (negative control) (*p* < 0.01) (**A**). The specific antisera against EtMIC3-bc1 protein significantly inhibited binding of the three phages to EtMIC3-bc1 protein compared with the irrelevant antibody against hexon protein of fowl adenovirus serotype 4 (FADV-4) (negative antibody control) (*p* < 0.01) (**B**). The phages A, D and W displayed specific binding with sonicated sporozoites protein. The binding of the three phages to recombinant EtMIC3-bc1 protein was used as positive control, and meanwhile the binding of unrelated phages from G protein of VSV to sporozoites was used as negative control to exclude the possibility of binding between any other phages and sporozoites protein (**C**). Each sample was tested in triplicate. Values represent mean ± SD. ∗ *P* < 0.05, ∗  ∗ *P* < 0.01.

All three target phages showed higher binding capability to EtMIC3-bc1 protein than unrelated control phages screened from G protein of VSV (*p* < 0.01). The three phages A, D and W diluted from 1 × 10^6^ pfu/100 μL to 1 × 10^12^ pfu/100 μL exhibited dose-dependent binding affinity with EtMIC3-bc1 protein (Figure 3A). The results of competitive ELISA showed that antisera against EtMIC3-bc1 protein diluted from 1:1500 to 1:1000 competitively inhibited binding of phages A, D and W to EtMIC3 protein. The irrelevant antisera against hexon protein of fowl adenovirus serotype 4 (FADV-4) did not exhibit dose-dependent inhibition (Figure 3B). The three phages A, D and W, especially phages A, showed higher binding capability to sporozoites proteins than negative control phages (*p* < 0.01) (Figure 3C).

### In vitro inhibition of sporozoites invasion

**Figure 4:**
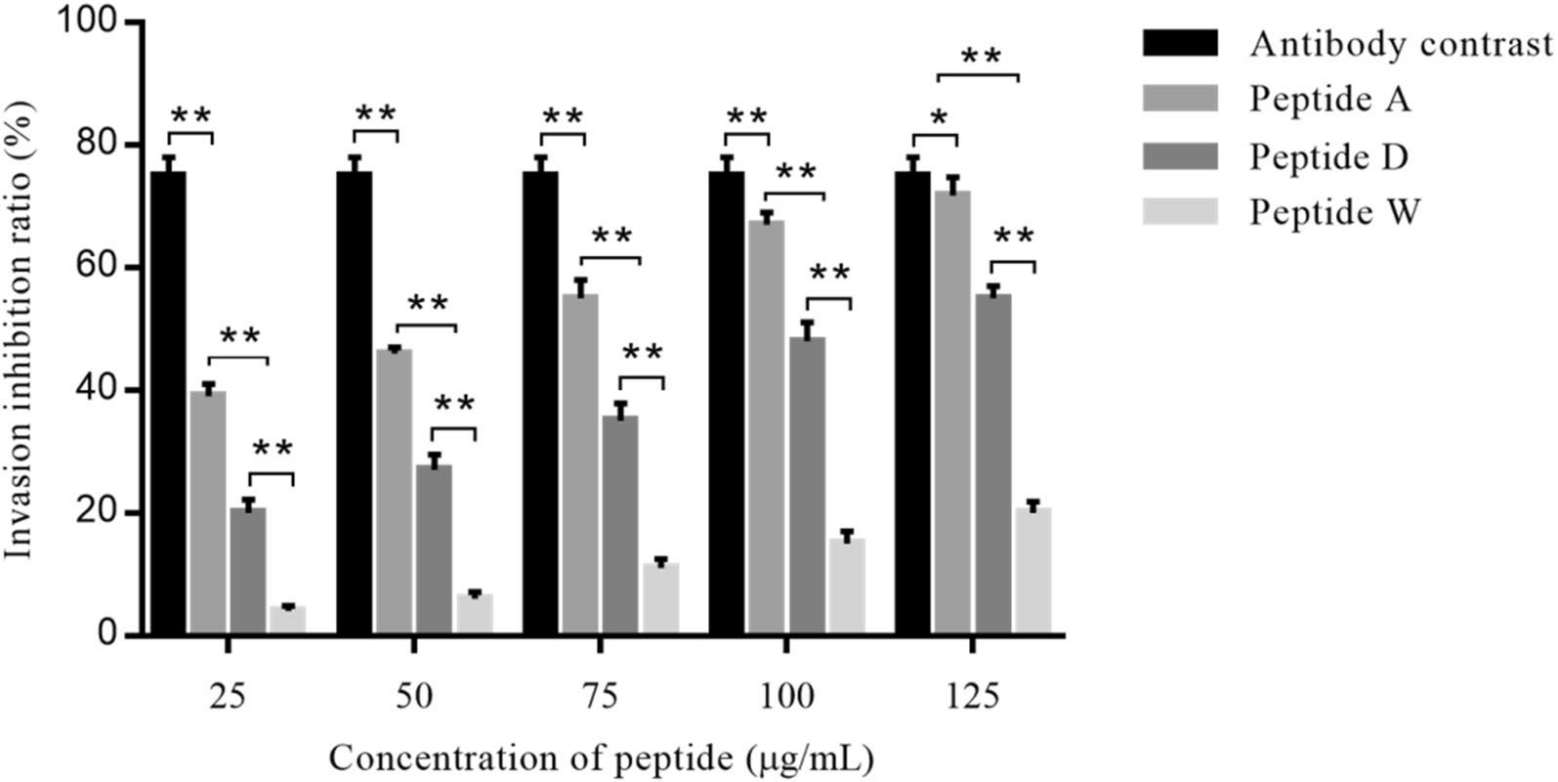
**Effect of three target peptides in inhibiting sporozoites invasion of cells in vitro.** The three target peptides, especially peptide A showed obvious effects in inhibiting sporozoites invasion of MDBK cells. The inhibition ratio showed in dose-dependent manners with concentraion of three peptides ranging from 25 to 125 μg/mL, showing inhibition ratio of 71.8% for peptide A, 54.6% for D and 20.8% for W at concentration of 125 μg/mL. 300 μg/mL of prepared polyclonal antisera against EtMIC3-b1c protein was used as positive control, showing inhibition ratio of 75.2%. The values represent mean ± SD (n = 5). ∗ *P* < 0.05, ∗  ∗ *P* < 0.01.

The maximum non-toxic concentration of the synthesized peptides A, D and W to MDBK cells was 125 μg/mL. The three peptides, especially A peptide, showed obvious inhibitory effects on sporozoites invasion of MDBK cells, and the inhibited ratio changed in dose-dependent manners with concentrations of peptides ranging from 25 μg/mL to 125 μg/mL. The observed highest inhibition ratio of 71.8% was presented by peptide A at concentration of 125 μg/mL, and 54.6% and 20.8% was observed for D and W peptide, respectively (Figure 4). The polyclonal antisera against EtMIC3-bc1 protein (300 μg/mL) prepared in this study was used as positive inhibitor for sporozoites invasion into cells, showing inhibited ratio of 75.2%.

### In vivo evaluation of anti-coccidial effects

**Figure 5:**
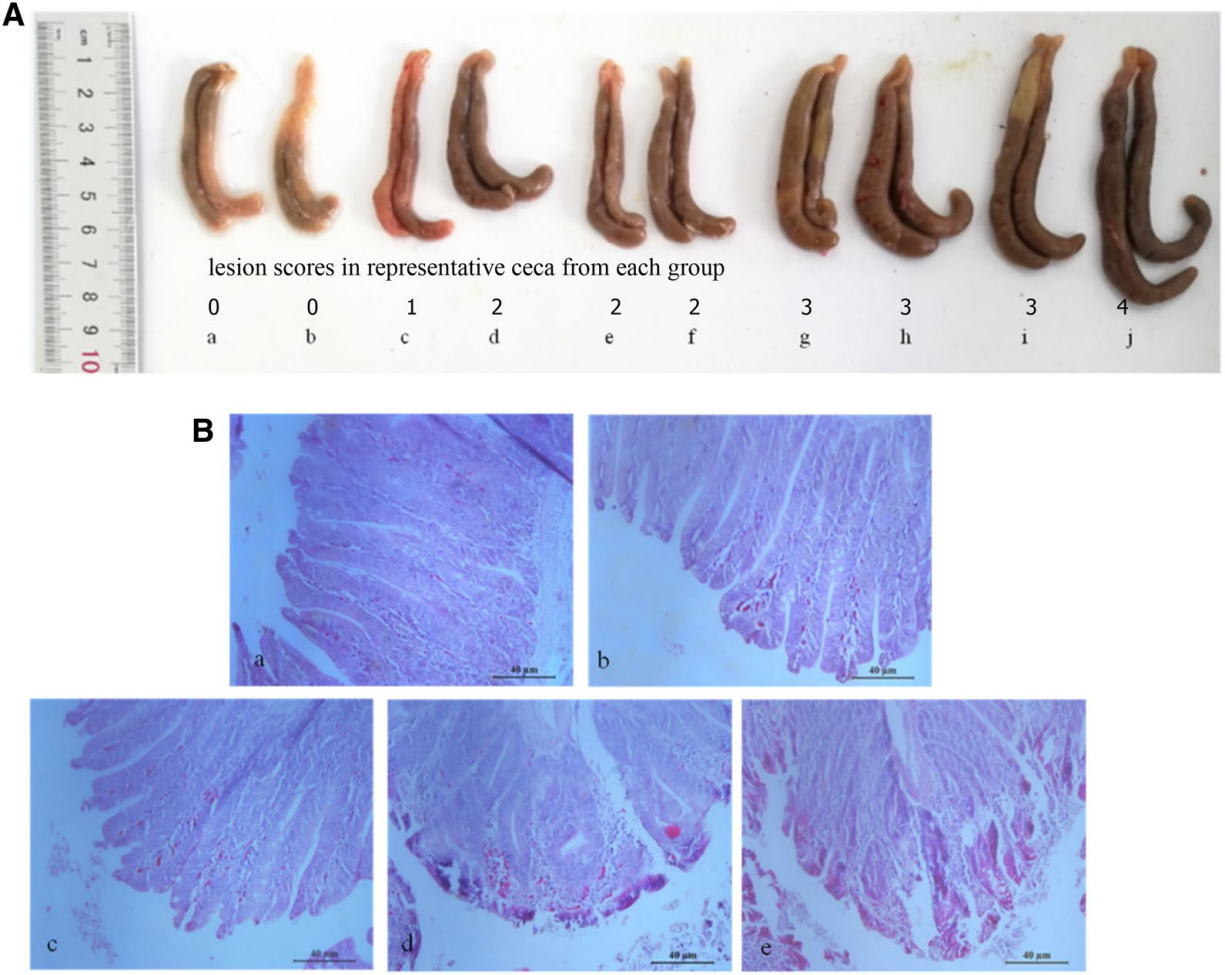
**Pathological changes in ceca of experimental chickens in each group.** On day 7 post infection (PI), chickens from challenged control group displayed severe typical gross pathological changes in ceca, including swelling and thickening, petechial hemorrhage on the serous membranes, and bloody cecal content (i and j, **A**), while chickens in the group orally fed with three phages A (c and d, **A**), D (e and f, **A**) and W (g and h, **A**), especially phages A and D, presented relatively moderate gross pathological changes. Chickens from unchallenged control group showed no gross pathological changes in ceca (a and b, **A**). The histopathological changes in cecal tissues of chickens in challenged control group were obvious, including compromised intestinal structural integrity, damaged intestinal villi, numerous red cells, and infiltration of inflammatory cells (e, **B**) (H.E. × 200), while chickens orally fed with phages A (b, **B**) (H.E. × 200), D (c, **B**) (H.E. × 200) and W (d, **B**) (H.E. × 200) presented relatively moderate histopathological changes in cecal tissues. Chickens from unchallenged control group showed no histopathological changes in cecal tissues (a, **B**) (H.E. × 200). Representative images were radomly selected from each group, and lesion scores in ceca were recorded by arabic numerals in (**A**).

As shown in Table 2, the weight gain of chickens orally fed phages A, D and W was higher than that in challenged control group (*P* < 0.05). The average cecal lesion scores of chickens in the group orally given phages A or D was significantly lower than that in the group with phages W and challenged control group (*P* < 0.01). The oocyst reduction ratio in the group orally fed phages A, D and W was 47.42%, 40.78% and 8.52%, respectively. On day 7 PI, chickens from challenged control group displayed severe typical gross pathological changes in ceca, including swelling and thickening, petechial hemorrhage on the serous membranes, and bloody cecal content (Figure 5A). The cecal tissues of chikens in challenged control group presented remarkable histopathological changes, including compromised intestinal structural integrity, damaged intestinal villi, numerous red cells, and infiltration of inflammatory cells, while ceca of chikens in the groups with three phages, especially with phages A or D presented mild gross pathological and histopathological changes (Figure 5B).

**Table 2:** **In vivo evaluation of anti-coccidial effects.**

Group	Challenge	Oral inoculation	Body weight gain	Relative body weight gain (%)	Average lesion score in cecum	Oocyst reduction ratio (%)
1	Unchallenged	PBS (pH7.2)	77.75 ± 2.46^a^	100	/	/
2	Challenged	Phages A	64.33 ± 3.27^b^	82.74	1.40 ± 0.34^A^	47.42%
3	Challenged	Phages D	60.08 ± 2.94^b^	77.27	1.57 ± 0.38^A^	40.78%
4	Challenged	Phages W	56.27 ± 3.85^b^	72.36	2.50 ± 0.46^B^	8.52%
5	Challenged	PBS (pH7.2)	46.04 ± 2.65^c^	59.21	3.10 ± 0.40^C^	0

21 day-old chicken was orally challenged with 1 × 10^4^ *E. tenella* sporulated oocysts. At days 0, 1 and 2 post challenge, each chicken was orally inoculated with 1 × 10^12^ pfu target phages. Significant difference between numbers with different capital letters (*P* < 0.01) and small letters (*P* < 0.05).

### Homology modeling and docking

**Figure 6:**
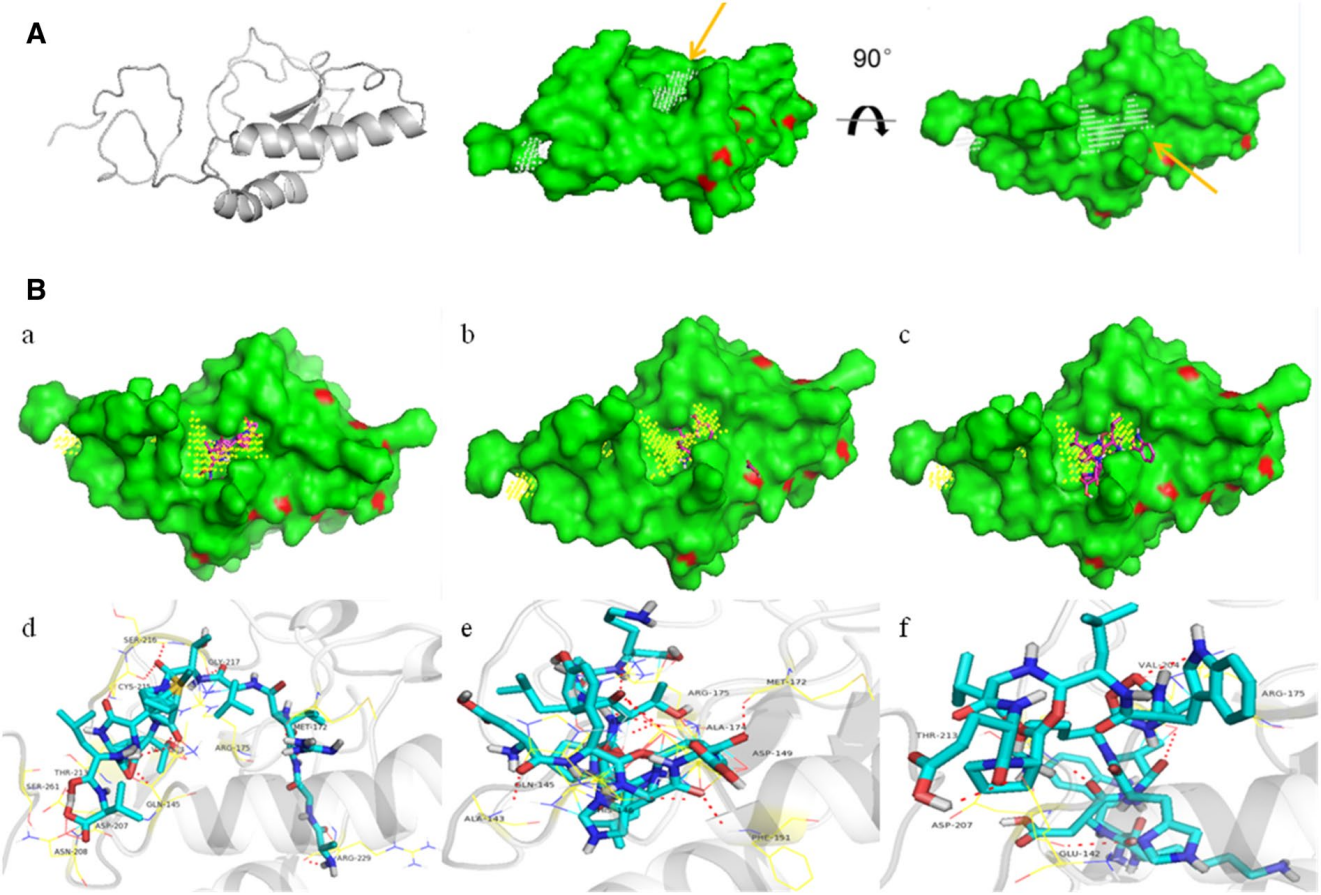
**Homology modeling and molecular docking between EtMIC3-bc1 protein and three target peptides. **Secondary and tertiary structure of EtMIC3-bc1 protein were constructed, and an obvious hydrophobic groove was observed in three-dimensional (3D) structure of EtMIC3-bc1 which is responsible for binding target peptides (showed by arrow) (**A**). Molecular docking between EtMIC3-bc1 protein and peptide A, D and W was analyzed, respectively. All twelve amino acids in peptide A completely insert into the hydrophobic groove of EtMIC3-bc1 protein, and twelve hydrogen-bonds were formed between amino acids in peptide A and in EtMI3-bc1 protein (a and d, **B**). For peptide D, partial amino acids insert into the hydrophobic groove of EtMIC3-bc1 protein, and nine hydrogen-bonds were observed (b and e, **B**). Only several amino acids in peptide W insert into the hydrophobic groove of EtMIC3-bc1 protein, and six hydrogen-bonds were formed (c and f, **B**).

The secondary and tertiary structure of EtMIC3-bc1 protein was repectively depicted, and an obvious hydrophobic groove was observed in three-dimensional (3D) structure of EtMIC3-bc1 which is responsible for binding target peptides (Figure 6A). The molecular docking between EtMIC3-bc1 protein and target peptides was analyzed, and intermolecular hydrogen-bonds were formed between amino acids in three target peptides and EtMIC3-bc1 protein (Figure 6B). The extent of binding between amino acids in the three peptides and EtMIC3-bc1 protein is different. All 12 amino acids in peptide A (a and d in Figure 6B), partial amino acids in peptide D (b and e in Figure 6B), and only several amino acids in peptide W (c and f in Figure 6B) inserts into the hydrophobic groove of EtMIC3-bc1 protein. The amino acids in peptide A, D and W contributing to the formation of hydrogen-bonds with amino acids in EtMIC3-bc1 protein are shown in Table 3.

**Table 3:** **Amino acids in EtMIC-bc1 protein and in peptide A, D and W that predicted for forming hydrogen-bonds.**

peptide	Hydrophobic amino acids	Amino acids for forming hydrogen-bonds in		Number of hydrogen-bonds	Common amino acids in peptide that contribute for forming hydrogen-bonds between peptides			
		peptides	EtMIC-bc1 protein		A and D	A and W	D and W	A, D and W
A	A^1^, L^4^, L^5^, M^9^, L^11^, V^12^	A^1^, R^3^, L^5^, T^6^ T^8^, S^10^, L^11^, V^12^	G^217^, S^216^, C^215^, V^204^, T^213^, R^175^, A^206^, D^207^, N^208^, Q^145^, R^229^, M^172^	12	Q^145^, M^172^, R^175^	V^204^, T^213^, D^207^	/	R^175^
D	Y^2^, L^7^, L^10^	D^1^, H^3^, D^4^, Y^2^, T^9^	M^172^, R^175^, A^174^, D^149^, F^151^, Q^145^, A^143^	9	Q^145^, M^172^, R^175^	/	R^175^	R^175^
W	W^1^, V^4^, A^7^, W^8^ L^9^, L^10^	D^3^, V^4^, H^5^, K^6^, A^7^, E^11^	V^204^, R^175^, T^213^, D^207^, E^142^	6	/	V^204^, T^213^, D^207^	R^175^	R^175^

## Discussion

Avian coccidiosis caused by *Eimeria* leads to serious ecomomical losses to the global poultry industry [23]. The development of alternative control strategies would be based on the deep understanding of the mechanism for *Eimeria* invasion of host epithelial cells. The lifecycle for *Eimeria* parasites includes several key developmental stages including sporozoites, merozoites, and gametocytes. Invasion for apicomplexan protozoan parasites was initiated based on the interplay between the apex of parasites and host cells, during which a series of secreted proteins at the parasite-host interface are indispensable for the parasites to penetrate into host cells. Apical membrane antigen 1 (AMA1) is one of the microneme proteins, and previous studies have shown that AMA1 protein secreted by sporozoites is important for invasion of *Toxoplasma godii* and *E. tenella* into host cells [14, 24]. Besides AMA1 protein, *E. tenella* microneme protein 3 (EtMIC3), one of the important invasion-related proteins released by secretory organelles existing in sporozoites, plays a crucial role in parasites attachment and subsequent invasion into host cells. It has been shown that EtMIC3 protein consists of seven microneme adhesive repeats (MARs), including four highly conserved internal repeats and three divergent external repeats [7, 25]. EtMIC3 contributes to guiding and binding sporozoites to the invasion site in chicken intestinal tract by recognizing BCL2-associated athanogene 1 (BAG1) and Endonuclease polyU-specific-like (ENDOUL) [26], and sialylated glycans [12, 13]. It was reported that AMA1-binding peptides effectively inhibited *T. gondii, P. falciparum and E. tenella* invasion into host cells [14, 27, 28]. However, the roles of peptide binding to EtMIC3 protein in inhibiting sporozoites invasion into host cells were not reported until now. In the present study, three phages (named A, D and W) with EtMIC3-binding capability were firstly identified. Analysis of amino acids in the three corresponding peptides revealed that peptide A, D and W appeared 8, 6, and 3 times, respectively, and 2 or 3 consecutive amino acids were similar between peptide A and D, and more hydrophobic amino acids were contained in peptide A, all of which suggesting that peptide A probably exhibited excellent functional characteristics in subsequent test. The ELISA results displayed obvious dose-dependent increase of binding capability between the three phages and EtMIC3-bc1 protein, which were reversely inhibited by polyclonal antisera against EtMIC3-bc1 protein in a dose-dependent manner. The above results demonstrated that the three phages, especially phage A specifically binds to EtMIC3-bc1 protein. Furthermore, the unrelated control phages showed lower binding capability to *E. tenella* sporozoites protein than the three phages, especially phage A and D (*p* < 0.01), further indicating that three target phages were EtMIC3-specific.

To detect the roles of three synthesized linear peptides in inhibiting sporozoites invasion of cells, the non-cytotoxic concentration of target peptides was determined to ensure survival of the labelled sporozoites under cell culture conditions. 125 μg/mL of peptides A, D and W effectively inhibited sporozoites invasion of cells, showing ratio of 71.8, 54.6 and 20.8%, respectively, which are consistent with the above analysis of peptide sequence and ELISA test.

To clarify whether the three phages provided anticoccidial effects in vivo, three phages were orally inoculated on day 0, 1, and 2 post homologous challenge. The results suggested that the three phages to some extent offered protection against *E. tenella* challenge. It was documented that the asexual developmental stage for *Eimeria* parasites on day 1 and 2 post infection of *E. tenella* sporulated oocysts was mainly sporozoites. Moreover, our preliminary animal experiment showed that the unrelated control phages did not show any anticoccidial effects. Therefore, the protections against homologous infection could be explained by the fact that the functional phages effectively bound to EtMIC3 protein secreted by *E. tenella* sporozoites in the intestinal tract, and then inhibited sporozoites invasion of cecal epithelial cells. These results and analysis were supported by previous report by Zuercher et al. [29], which showed that the functional phages retained activity in the gastrointestinal environment.

The molecular docking between target peptides and the tertiary structural model of EtMIC3-bc1 protein revealed that binding between the three peptides and EtMIC3-bc1 protein was coordinated by hydrogen-bonds. The three amino acids Q^145^, M^172^ and R^175^ in EtMIC3-bc1 protein that are responsible for forming nine hydrogen-bonds with peptide D, and the three amino acids V^204^, T^213^ and D^207^ in EtMIC3-bc1 protein that are responsible for forming six hydrogen-bonds with peptide W were all contained in the twelve amino acids G^217^, S^216^, C^215^, V^204^, T^213^, R^175^, A^206^, D^207^, Asn^208^, Q^145^, R^229^, M^172^ that are responsible for twelve hydrogen-bonds with peptide A. The above analysis provides the probable explanations for the function of 3 peptides in vitro and in vivo.

Periz et al. [11] and Lai et al. [12] reported that EtMIC3 specifically recognized and bound to α 2,3-linked sialic acid that predominantly distributed in ceca, which to some extent explained the tissue tropism of *E. tenella*. Moreover, Lai et al. [12] reported that competitors of EtMIC3-sialic acid binding inhibited sporozoites invasion of MDBK cells, which is consistent with the results in the present study that sporozoites invasion of MDBK cells was inhibited by EtMIC3-binding peptides. Cowper et al. [13] demonstrated that some amino acids in type I and II MAR (microneme adhesive repeat) from *T. gondii* microneme protein 1 (TgMIC1), *T. gondii* microneme protein 13 (TgMIC13), *N. caninum* microneme protein 1 (*N*cMIC1), and EtMIC3 are conserved. Based on the above reports, in the present study, it is interesting for us to find that five aimino acids Q^145^, R^175^, D^207^, C^215^ and G^217^, two amino acids Q^145^ and R^175^, and two amino acids D^207^ and R^175^ contained in EtMIC3-c1(MARc1) protein contributed for forming hydrogen-bonds with peptide A, D and W, respectively, and meanwhile all the above amino acids are also conserved among TgMIC1, TgMIC13, NcMIC1 and EtMIC3, suggesting that the three peptides, especially peptides A, probably to some extent displayed inhibition of *T. gondii* and *N. caninum* invasion of host cells.

## Supplementary Information

**Additional file 1. SDS-PAGE analysis of EtMIC3-bc1 protein expressed in** ***E. coli*** **BL21 cells.** A band of 41 kDa corresponding to EtMIC3-bc1 protein was observed. M, Protein molecular weight marker. Lane 1, Recombinant positive bacteria without induction by isopropyl-b-D-thiogalactopyranoside (IPTG) (negative control). Lane 2–5, EtMIC3-bc1 protein expressed in *E. coli*. BL21 cells induced by IPTG for 0, 1, 2 and 3 h, respectively. Lane 6 EtMIC3-bc1 protein purified by affinity chromatography with Ni-conjugated Sepharose.

**Additionnal file 2. Detection of prepared anti-EtMIC3-bc1 polyclonal antisera by Western blot.** Sporozoites protein and recombinant EtMIC3-bc1 protein samples were respectively separated by SDS-PAGE, then transferred to nitrocellulose membranes. The prepared rabbit anti-EtMIC3-bc1 polyclonal antisera specifically recognized target proteins, showing band of 41 kDa. Lane M, Protein molecular weight marker. Lane 1, Band of recombinant EtMIC3-bc1 protein expressed in *E. coli* BL21 cells. Lane 2, Band of EtMIC3 protein in sporozoites.

## Data Availability

Not applicable.

## Abbreviations

AMA1: apical membrane antigen 1
CFDA-SE: carboxyfluorescein diacetate succinimidyl ester
ELISA: enzyme-linked immunosorbent assay
EtMIC3: *E. tenella* Microneme protein 3
HRP: horseradish peroxidase
IPTG: isopropyl-b-D-thiogalactopyranoside
MARR: microneme adhesive repeat regions
SPF: specific pathogen-free
3D: three-dimensional
