# Supplementary figures and images for: Specific EtMIC3-binding peptides inhibit Eimeria tenella sporozoites entry into host cells
Source: Vet Res. 2021 Feb 17;52:24. doi: 10.1186/s13567-020-00873-y (PMC7888181; doi:10.1186/s13567-020-00873-y)

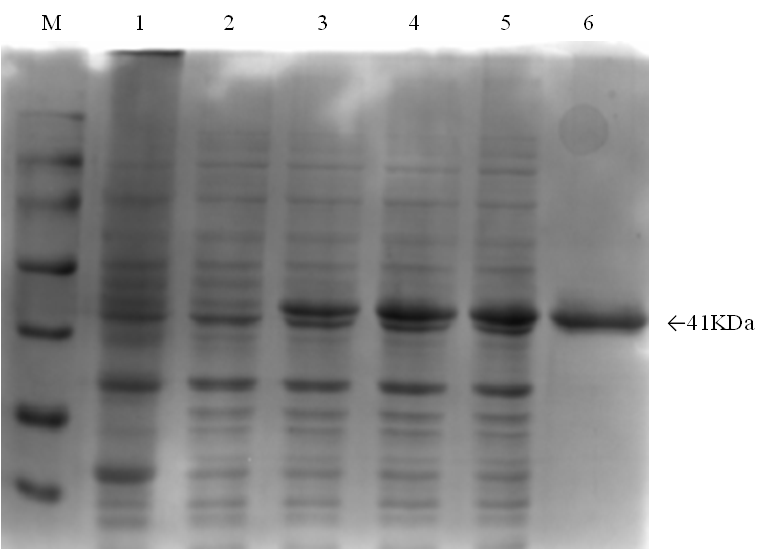

Supplement: Supplementary file 1 — Additional file 1. SDS-PAGE analysis of EtMIC3-bc1 protein expressed in E. coli BL21 cells. A band of 41 kDa corresponding to EtMIC3-bc1 protein was observed. M, Protein molecular weight marker. Lane 1, Recombinant positive bacteria without induction by isopropyl-b-D-thiogalactopyranoside (IPTG) (negative control). Lane 2–5, EtMIC3-bc1 protein expressed in E. coli. BL21 cells induced by IPTG for 0, 1, 2 and 3 h, respectively. Lane 6 EtMIC3-bc1 protein purified by affinity chromatography with Ni-conjugated Sepharose. [file 13567_2020_873_MOESM1_ESM.doc]

**
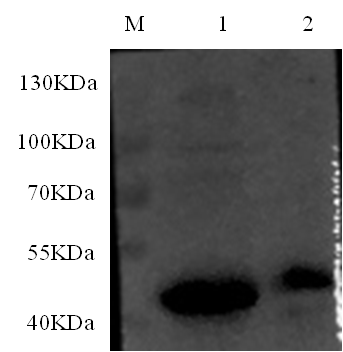
**

Supplement: Supplementary file 2 — Additionnal file 2. Detection of prepared anti-EtMIC3-bc1 polyclonal antisera by Western blot. Sporozoites protein and recombinant EtMIC3-bc1 protein samples were respectively separated by SDS-PAGE, then transferred to nitrocellulose membranes. The prepared rabbit anti-EtMIC3-bc1 polyclonal antisera specifically recognized target proteins, showing band of 41 kDa. Lane M, Protein molecular weight marker. Lane 1, Band of recombinant EtMIC3-bc1 protein expressed in E. coli BL21 cells. Lane 2, Band of EtMIC3 protein in sporozoites. [file 13567_2020_873_MOESM2_ESM.doc]
